# How Do Review Party's Identity Cues Influence Consumers' Online Review Adoption Intention?

**DOI:** 10.3389/fpsyg.2022.865877

**Published:** 2022-07-15

**Authors:** Liang Xiao, Fujun Wang, Shu Wang, Fumao Yu, Yan Wang

**Affiliations:** ^1^School of Management and E-Business, Zhejiang Gongshang University, Hangzhou, China; ^2^Modern Business Research Center, Zhejiang Gongshang University, Hangzhou, China

**Keywords:** review party's identity cue, adoption intention, N2, LPP, event-related potential, neuromanagement

## Abstract

Professionalism and popularity are two important external identity cues of the review party. Previous studies have mostly focused on the content of the reviewers' comments. However, few studies have explored the potential impact of the review party's cues on consumers' adoption willingness and consumption behavior. This study mainly examined the neural mechanisms of how the differences in the two identity cues of the review party affect consumers while adopting the comments. The current study employed an event-related potential (ERP) experiment, in which the participants were asked to make a personal choice quickly based on the review party's identity cues after seeing the target product. A 2-level professionalism (low vs. high) x 2-level popularity (low vs. high) experiment design was used to test the impact of the review party's professionalism and popularity on consumers' intentions to adopt the review. The behavioral data reveal that the two identity cues of the review party impact the adoption rate, and the review party's popularity has an impact on the reaction time. The ERP data indicate that the review party's popularity affects the perceived risk (the N2 component, which is a high-risk signal) and the two identity cues of the review party affect the evaluation and classification process [the later positive potential (LPP) component]. These results indicate that when the review party has a high degree of professionalism, its popularity has less influence on consumers' review adoption intention. On the contrary, when the level of professionalism is low, high popularity will promote consumers' review adoption intention. Compared to professionalism, popularity is a higher risk cue for consumers.

## 1. Introduction

Cue utilization theory identifies cues as a series of product-feature-related information that can help consumers determine product quality. Cues can be classified into internal and external cues based on consumers' perceived product quality. Internal cues reflect the product's physical characteristics and are directly connected to product quality. External cues have no direct connection to the product itself but can aid consumers in inferring product quality, such as brand and packaging (Rao and Monroe, 1988). Consumers tend to rely on internal cues if obtaining and confirming internal cues are affordable. Otherwise, they will turn to external cues. However, the complexity of the real-life online shopping environment prevents consumers from easily obtaining and evaluating the internal cues of the products. It makes thorough evaluations of products like in the offline shopping scenario impossible. The risk and uncertainty are thus increased for online shopping consumers. Therefore, customers need to rely on external cues to make decisions in the online shopping scenario, and product review is among the many important external cues (Utz et al., 2012).

The rapid development of e-commerce has made product reviews a significant source for consumers to learn product information. Reviews by different parties tend to judge the same product differently, which brings difficulties for customers to identify the genuineness and quality of the product review as consumers lack product knowledge. Fortunately, the information of the reviewers (reviewer's identity cues) comes to the consumers' aid. Previous studies have found that reviewers' identity cues significantly correlate to consumers' purchase intentions based on the polarity of the review (Li and Liang, 2021). Additionally when the reviewers are experts or disclose their identity, their reviews are more likely to be helpful (Filieri et al., 2019). Therefore, the identity cues of the review parties (reviewer) act as important reference factors for consumers to decide whether to adopt the review (Ying, 2012; Guo and Zhou, 2016). Studies reveal that different identity cues of review parties influence audiences' intentions to adopt their reviews (Hovland and Weiss, 1951; Lee and Youn, 2009; Gu et al., 2012). Identity cues help consumers evaluate product quality, reduce purchase risk, and increase purchase intentions by influencing the perceived value and perceived quality of the products. Current studies lack the investigation and examination of the underlying psychological mechanism of review parties' identity cues influencing consumers' adoption intention. To reveal the mechanism, we employ the Event-Related Potentials (ERPs) technology in this article to explore the impact of different review parties' identity cues on consumers' intentions to adopt the product reviews.

Recent studies have found that professionalism, authority, normalization, and independence of the review parties significantly impact consumers' intention to adopt reviews (Shang et al., 2017). Studies have found that the review party's expertise (professionalism) positively affects review helpfulness (Yang et al., 2019). Compared with product reviews published by ordinary users, professional reviews provided by experts provide consumers with more authoritative judgments through a certain degree of personal bias, and the information they share will be more easily accepted and trusted by consumers (Bansal and Voyer, 2000; Zeng, 2011). For example, judging the rank of the review parties' professionalism indirectly affects consumers' purchase intention (Amblee and Bui, 2007; Chang et al., 2012; Sun et al., 2020). The professional knowledge and influence of the review parties attract more consumer attention (Guo and Zhou, 2016; Han, 2020). In addition, high-popularity review parties greatly affect the consumers' opinions on certain products and consumers' purchase decisions. Their reviews can reach consumers and be advertised more quickly with the help of social networks and we-media (Jin and Phua, 2014; Knoll and Matthes, 2016; Hwang and Zhang, 2018). Early studies have attributed the essence of popularity affecting consumer decision-making to the binding of fan economy and consumption habits, which create a strong stickiness between review parties and their fans, and induce fans' consumption behaviors (Zhong et al., 2020). High popularity review parties are more influential and have stronger advertising effects, which lead to the active consumer following, the imitation of information shared by high popularity review parties, the building of consumer confidence, and the reduction of decision-making uncertainty (Dean and Hal, 1999; Luan et al., 2019). Some studies reveal the persuasiveness of the reviews can be reflected by the identity cues of the review parties. For example, the innovative ability (Yue and Zhang, 2011), the number of followers (Hong et al., 2017), the rating (Park and Lin, 2020), the gender (Shin et al., 2017) of the review parties, and the frequency and amount of information posted by the review parties (Forman et al., 2008; Hernández-Ortega, 2017). To sum up, the professionalism and popularity of the review parties are the two identity cues that consumers care for most. They influence consumers' intentions to adopt the product reviews.

Different from previous studies on adoption intentions which use mainly traditional methods of self-reporting with an unsolved challenge of exploring related issues while consumers are unable or unwilling to express their feelings or preferences fully (Dimoka et al., 2012). Cognitive neuroscience researches indicate that more than 90% of the information is processed by the subconscious mind of the brain, but this subconscious information processing plays a critical role in consumer decision-making (Ozkara and Bagozzi, 2021). ERPs technology possesses the advantages of high time resolution and non-invasiveness, providing a new method and approach for studying the process of the subconscious brain cognitive activity (Wang et al., 2021). Wang et al. found that product ratings significantly affect consumers' risk perception with ERPs technology, while the combination of high ratings and low sales causes significant cognitive conflicts among consumers (Wang et al., 2016). According to Kotler's stimulus-response model, identity cues as external stimuli affect consumers' psychological activities, and the perception and diagnosis of information will ultimately affect consumers' intentions and behaviors (Barich and Kotler, 2016). Previous studies have shown that the ERP component of N2 generally has a high correlation with cognitive conflict and risk perception (Folstein and Petten, 2008). The difficulty and conflict of consumers' decision-making will be increased as consumers perceive higher risks. Consumers need more cognitive control to make a decision (Chen et al., 2012), which was manifested by an increase in the amplitude of the N2 component (Ridderinkhof et al., 2004; Ma et al., 2015; Wang et al., 2016). The N400 component reflects semantic conflict and information conflict (Wang et al., 2016). Studies have found that N400 may also reflect other non-semantic conflicts, such as cognitive conflicts and emotional conflicts (Qiu et al., 2007; Steffensen et al., 2008; Taake et al., 2009). The later positive potential (LPP) component presents in the later stage of decision-making evaluation and classification processing (Ito and Cacioppo, 2000; Schupp et al., 2010; Herring et al., 2011; Ryoichi et al., 2014). Classification of stimuli in the evaluation dimension induces significant LPP amplitudes (Ito and Cacioppo, 2000; Ryoichi et al., 2014). Neuromarketing research shows an obvious evaluation stage before consumers make the final purchase decision. The LPP amplitude change is closely related to the stimulus of the final evaluation classification (Wang et al., 2016). Therefore, from the perspective of risk perception and choice assessment, this article uses N2, N400, and LPP components to analyze and study the underlying psychological process of review parties' identity cues influencing consumers' intentions to adopt the product reviews.

This study constructs a 2-level professionalism (low vs. high) x 2-level popularity (low vs. high) experiment and assumes that there are differences in consumers' responses to review parties' identity cues: high-professionalism and high-popularity review parties may be favored most, while low-professionalism and low-popularity review parties may be least favored. Consumers often find it more difficult to make judgments and decisions when faced with conflicting identity cues of the review parties. In the final evaluation and classification stage, consumers tend to choose external information that is beneficial to them more rationally. Considering consumers' risk perception of the identity cues of review parties, the hypothesizes of this study are:

**H1:** Low-professionalism or low-popularity induces higher amplitudes of the N2 component.
**H2:** Conflict identity cues produce greater N400 amplitudes.
**H3:** Before making the final decision on the intention to adopt, consumers make decisions for rational use of information, leading to greater and longer LPP amplitudes.

## 2. Methods

### 2.1. Participants

A total of 16 healthy subjects (7 men, 9 women) were recruited for this experiment, ranging in age from 20 to 30 years old (Mean = 23.625, *SD* = 1.708). All subjects are right-handed with normal or corrected-to-normal vision. The subjects reported no mental illness or any history of neurological disorders. The subjects signed written informed consent before the experiment and were paid 50 CNY after the experiment. The subjects were reported to have read product reviews on e-commerce platforms within the last month and had a good rest the day before the experiment. During the experiment, subjects were asked to stay conscious and focused.

### 2.2. Experimental Materials

The design of the experimental materials was conducted in three phases: stimulus materials selection, identity cue extraction, and stimulus material emotion validation.

#### 2.2.1. Stimulus Material Selection

The experiment chose electronic products as the experimental stimulus material mainly because these products are generally functional. Unlike the observable individual differentiation of hedonic products, electronic products can minimize the influence of individual preference on decision intention. In addition, electronic products are generally complex, and consumers need specialized knowledge to buy such products, which leaves room for manipulating review parties' identity cues. Most of the subjects participating in this experiment are college students who are familiar with electronic products and have a high frequency of purchase, which can help the subjects enter the experimental situation more quickly.

#### 2.2.2. Identity Cue Extraction

**Figure 1:**
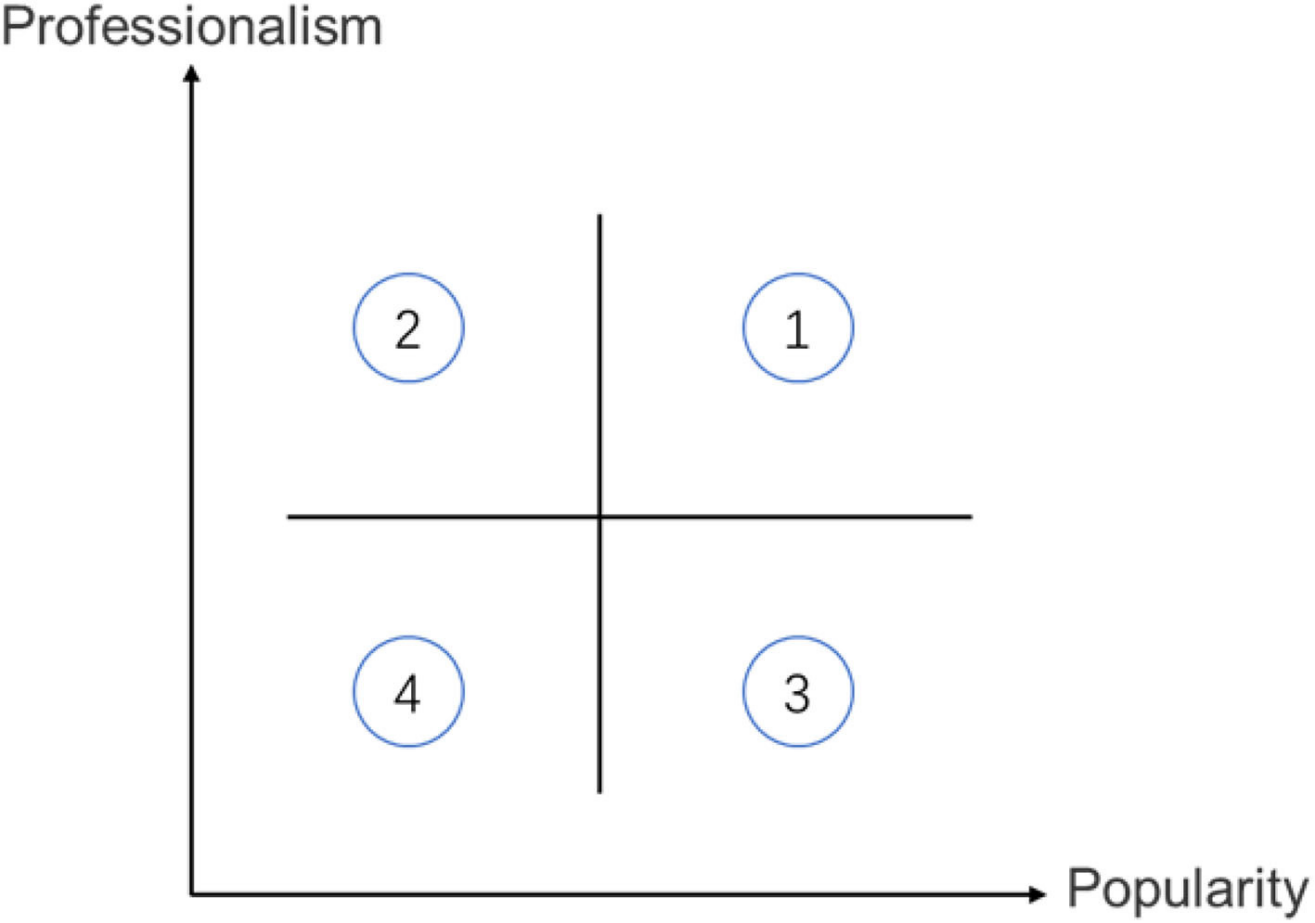
Schematic diagram of the classification of review parties.

To naturally simulate the situation of consumers browsing the product reviews and enhance the effectiveness and objectivity of the experiment. The experiment refers to the identity cues of the review party from the real-life scenario. We appropriately processed the information and data of the review party, removed anything that might cause extraordinary perceptions of the subjects, and made all the other contents of the stimulus materials except the identity cues identical. Referring to previous studies on reviewer information, we employed the classification method conceived by Friedman and others: stars and celebrities, experts, and typical consumers (Friedman et al., 1979) in our study. The two important identity cues of the review party's professionalism and popularity are then extracted and classified as: 1. high professionalism and high popularity, 2. high professionalism and low popularity, 3. low professionalism and high popularity, and 4. low professionalism and low popularity. The extracted identity cue combinations are illustrated in Figure 1. The four combinations correspond to four types of review parties with different identity cues in real life: experts, review agencies, Internet celebrities, and ordinary users and enthusiasts.

#### 2.2.3. Stimulus Material Emotion Validation

**Figure 2:**
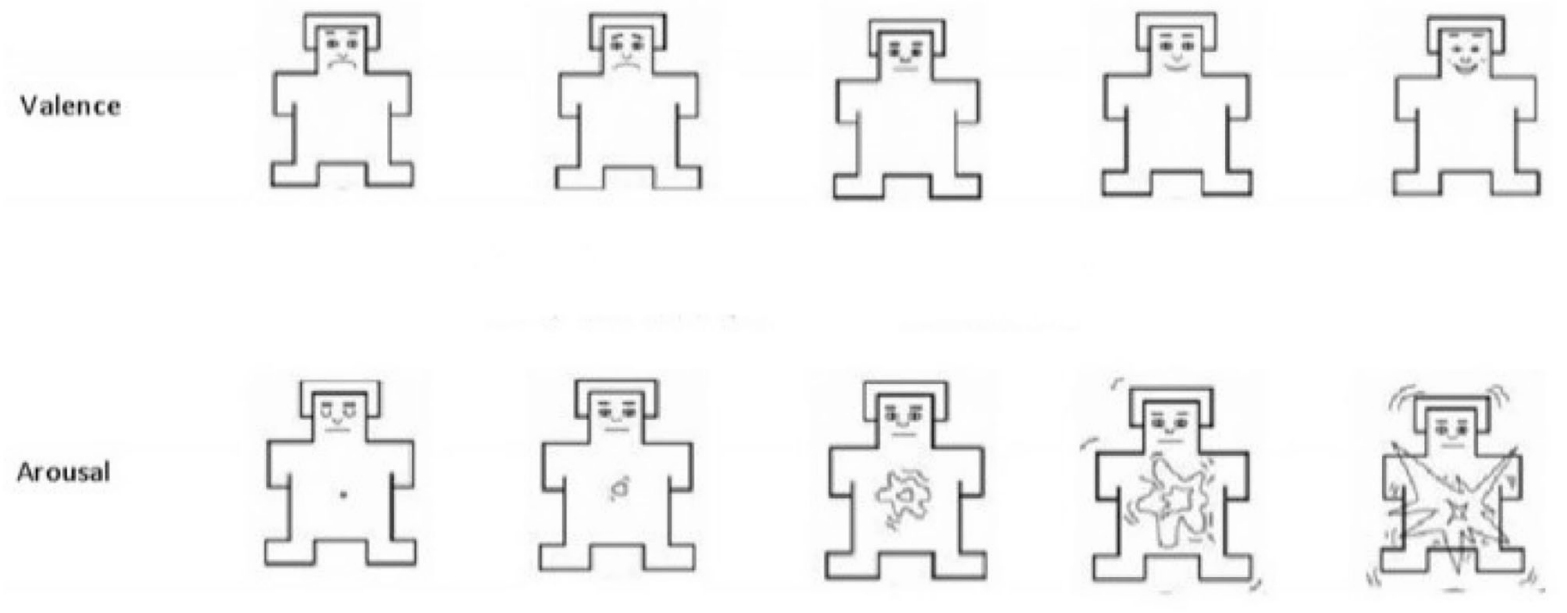
Improved SAM questionnaire.

Self-Assessment Manikins (SAM) is a nonverbal and picture-oriented emotional self-assessment tool designed and proposed by Lang et al. It can be used to directly measure a person's emotional response to various stimuli by the degree of pleasure (Valence), arousal (Arousal), and dominance (Lang et al., 1980; Hodes et al., 1985). According to the actual needs of the research, we tailored the standard SAM questionnaire, considering the two aspects of valence and arousal, as shown in Figure 2. In the tailored SAM questionnaire, valence ranges from a crying face to a smiling face (1–9), and arousal ranges from a sleepy face with eyes closed to an excited face with eyes opened, and the degree of arousal gradually rises.

**Figure 3:**
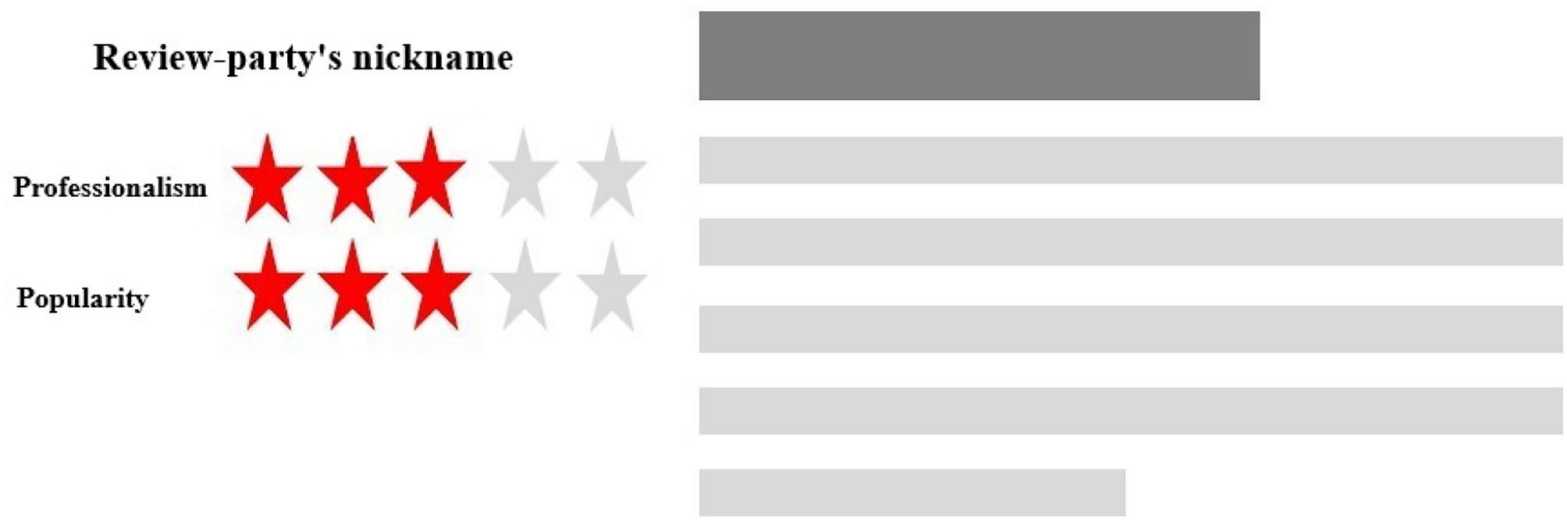
Sample stimulus material for a review.

Real-life product review contents tend to be versatile. Reproducing real-life product reviews in the experiment can be distracting as our study focuses on the review party's identity cues. It is necessary to preprocess the review content to avoid the stimulus materials from bringing excess impacts on subjects' psychological and emotional states. The review content on the stimulus materials should leave enough room for the subjects to focus on the review party's identity cues and grasp the whole idea of the review. The contents were designed into “gray bars,” referring to the popular “placeholder” practice in e-commerce App prototyping and content loading designs. An example stimulus material is depicted in Figure 3, which illustrates a review party's two identity cues of professionalism and popularity with its review content of an electronic product. The SAM questionnaire results showed that the stimulus materials raised no excessive emotional response. The mean valence was 4.713 (*SD* = 1.312), and the mean arousal was 4.635 (*SD* = 1.106).

### 2.3. Experiment

#### 2.3.1. Experimental Design

**Figure 4:**
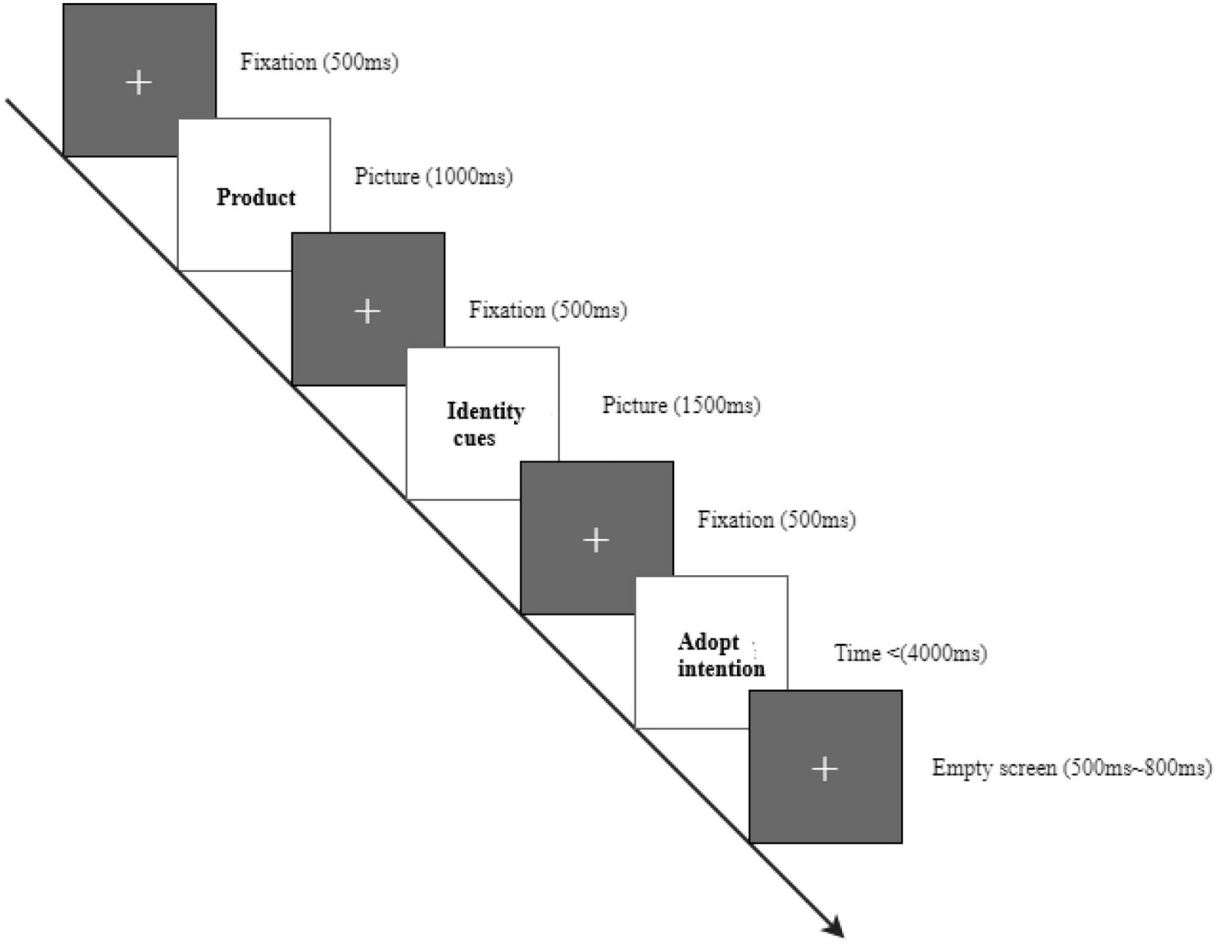
The trial sequence (Subjects are instructed to choose their intentions to adopt the review according to the different identity cues).

We designed the experiment as 2-level professionalism (high vs. low) x 2-level popularity (high vs. low). We scraped review party profiles from common e-commerce Apps and removed personal information, brand identity, and other information that could be used to target a specific review party. Review parties with neutral professionalisms and popularities were removed. The rest data were then tuned to generate the materials matching the four combinations of the experiment design. The images of the materials were processed to be uniform in size (450 x 550 px), hue, brightness, and saturation. Each experiment trail begins with a “+” sign lasting 500 ms to focus subjects' visual attention. Then a product image appears for 1,000 ms, followed by another “+” sign that lasts for 500 ms. The review with the review party's identity cues is then shown up for 1,500 ms, followed by another “+” sign that lasts for 500 ms. The subject is then prompted with a 5-level Likert scale to choose from their adoption intention using the left mouse button. After the subject finishes choosing, an empty screen shows up for 500–800 ms. The trail sequence of the experimental design is illustrated in Figure 4.

#### 2.3.2. Experimental Settings

The experiment was conducted in a 2 x 2 m room. The room is sound-proof and radio-proof with artificial illumination. The subject was asked to sit comfortably in front of a 21-inch computer monitor (with a resolution of 1,280 x 1,024). The distance between the subject and the monitor was around 80 cm. An electrode cap was put on the subject's scalp to record the EEG signal. The subject used a wired mouse to respond to the experiment. Testers explained the experiment contents and guided the subject through some training trails before starting the main experiment. The experiment instruction appeared on the computer monitor before the main experiment would ask the subject to consider browsing an e-commerce App and purchasing based on the review parties' identity cues. The product images and the identity cues were selected randomly in each trial to prevent subjects from predicting the next trial. A total of 220 trials were conducted during the experiment. The E-prime 3.0 software was used to present the experiment and collect the subjects' behavioral data.

#### 2.3.3. EEG Recordings and Analyses

The EEG signal of the experiment was recorded by a Neuroscan Synamp2 amplifier connecting a 64-channel electrode cap driven by the Neuroscan Curry 8 software. The reference electrode (REF) and ground electrode (GND) were connected. The skin of the left supraorbital and infraorbital ridges of the left eye and the lateral sides of both the left and right eye canthi were treated with a scrub. Electrodes were placed onto these skin areas to record the vertical and horizontal eye movement, respectively. The skin where the electrodes were attached was treated with a scrub. Other electrodes were F3, Fz, F4; C3, CZ, C4; P3, Pz, and P4. The electrode connection conductivity was adjusted to below 5Kω by injecting conductive paste between the electrodes and the skin. The sampling rate of the recording was 100 MHz, and a 0.10–100 MHz band-pass filter was applied to the recording.

The EEG data were analyzed offline with EEGLAB13.6 run on Matlab 2020b. The main phases were: re-referencing, resampling, filtering, and artifact removal. The bilateral left and right mastoids (M1, M2) were used as offline reference electrodes. The EEG data were resampled to 500 Hz with data exceeding ±80μV removed. An 0.1–30 Hz band-pass filter was applied to the offline EEG data. The ICA was used to remove eye movement artifacts. The EEG data were then segmented by a 1,000 ms time window (200 ms pre-trigger before the stimulus onset to 800 ms post-trigger after the stimulus onset). The ERP components were then extracted by averaging the EEG segments adhering to their corresponding stimuli. The N2 component from the frontal lobe, the N400 component from the central area, and the LPP component from the parietal lobe were extracted and analyzed. Statistical analyses of repeated measurements ANOVA were then conducted on the ERP components data and the subjects' behavioral data with SPSS 25.0 software. Simple effect analysis and *post-hoc* multiple comparisons were used in case of an interaction effect between factors.

## 3. Results

### 3.1. Behavioral Results

**Figure 5:**
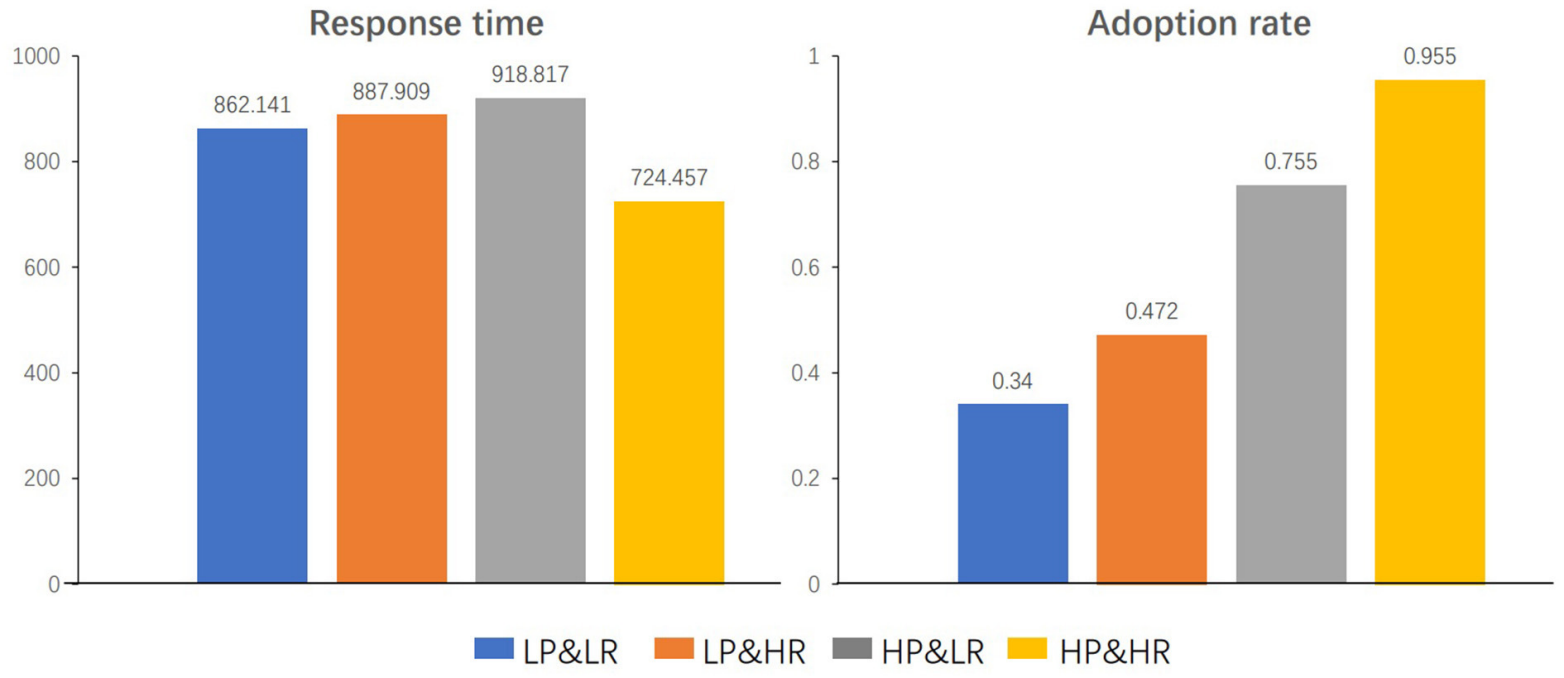
Response time **(right)** and adoption rate **(left)** under different conditions.

The behavioral data of this experiment mainly includes two parts: response time and adoption rate. The response time indicates the length of time from viewing the product information to responding to the experiment. That is the shortest time the subject receives the stimuli and performs cognitive processing to make adoption decisions. The adoption rate refers to the percentage of adoptions made under different stimulus types. That is the proportion of intentions after being stimulated by the four identity cues [the adoption intention ranges from low(1) to high(5)]. Table 1 shows the average response time (RTs, Response time) and mean adoption rate (Ars, Adoption rate) of the subjects under different review parties' identity cues. Figure 5 illustrates the comparison of the adoption rates and response times under different conditions, respectively.

**Table 1:** Mean adoption rate and mean response time across four conditions.

**Condition**	**Adoption**	**Response time**	**SD of**
	**rate (Ars)**	**(RTs) (ms)**	**response time**
C1:LP&LR	0.340	862.141	256.708
C2:LP&HR	0.472	887.909	318.588
C3:HP&LR	0.755	918.817	387.833
C4:HP&HR	0.955	724.457	173.215

*HP, high professionalism; LP, low professionalism; HR, high reputation; LR, low reputation*.

A 2 × 2 within-subjects repeated measures ANOVA showed that the main effects of professionalism [*F*_(1,13)_ = 169.706, *p* < 0.001] and popularity [*F*_(1,13)_ = 28.921, *p* < 0.001] on the adoption rate are significant; the interaction effect of professionalism and popularity is not significant [*F*_(1,13)_ = 0.691, *p* = 0.421].

Meanwhile, results of a 2 x 2 repeated measures ANOVA showed that popularity has a significant main effect on Response time [*F*_(1,13)_ = 5.047, *p* < 0.001] while professionalism does not demonstrate such an effect [*F*_(1,13)_ = 1.778, *p* = 0.205]. The paired sample *t*-test shows that under high professionalism, high and low popularity correlates with response time (*P* < 0.005), while no significant correlation was shown under low professionalism (*P* = 0.249).

### 3.2. ERP Results

#### 3.2.1. N2

**Figure 6:**
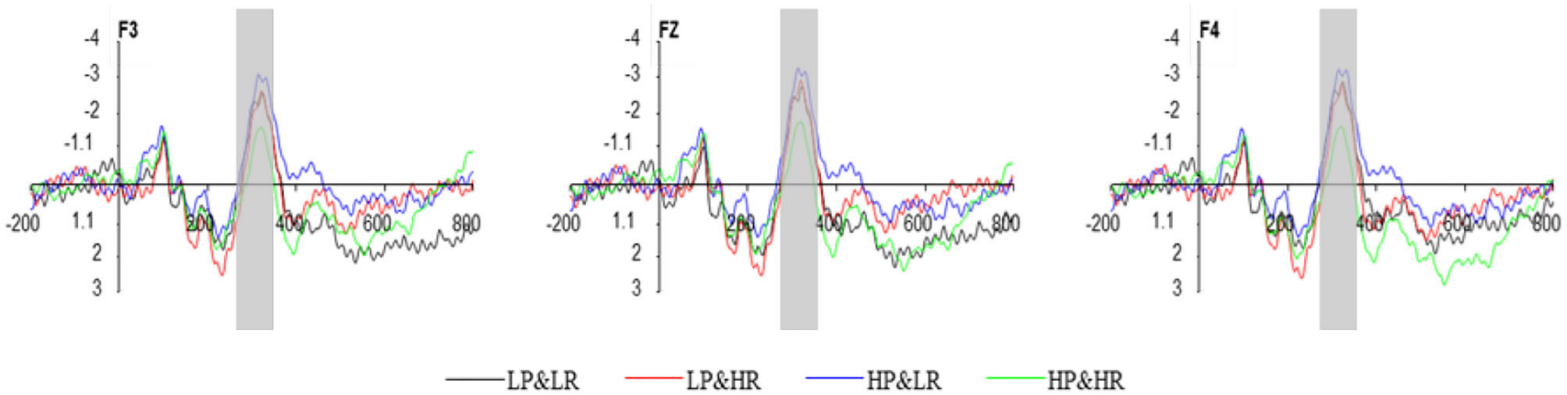
The average waveform of N2 component at F3, Fz, and F4.

Two of the subjects' ERP data were removed from the analysis due to recording errors. ERP and behavioral data of the rest 14 subjects were analyzed. The N2 component usually appears in the frontal lobe. We performed a 2 (HP vs. LP) x 2 (HR vs. LR) x 3 (frontal electrode sites: F3, Fz, F4) within-subjects repeated measures ANOVA on ERP amplitudes between 310 and 330 ms (Figure 6). The results showed that the main effect of professionalism is not significant [*F*_(1,13)_ = 0.961, *p* = 0.345], and the main effect of popularity is significant [*F*_(1,13)_ = 6.937, *p* = 0.021], the interaction effect between professionalism and popularity is not significant [*F*_(1,13)_ = 0.688, *p* = 0.422]. As hypothesized, N2, associated with risk perception, is elicited in the adoption decision-making process. *Post-hoc* multiple comparisons show that low popularity (C1: –3.480 and C3: –3.577) N2 amplitude is greater than high popularity (C2: –2.967 and C4: –2.199) N2 amplitude, which means that under low popularity conditions, the perceived risk is higher. Therefore, in the early stage of perceived risk, the popularity identity cue of the review party had an impact on the amplitude difference of N2. The different N2 amplitude indicates that there is an early stage of risk perception before the subjects decide to adopt. Thus, H1 is partially supported.

When there are different overall amplitudes under the two conditions, the interaction of factors involving electrode position has no clear meaning (Mccarthy and Wood, 1985). The data were normalized to eliminate the overall amplitude difference between different conditions to determine whether the interaction between the experimental conditions and the electrode sites reflects the actual internal source difference. The results once again proved that the normalization process is invalid (Urbach and Kutas, 2002). Therefore, in most cases, only a brief report on the observed electrode site interaction is required without further explanation (Luck, 2014).

#### 3.2.2. N400

**Figure 7:**
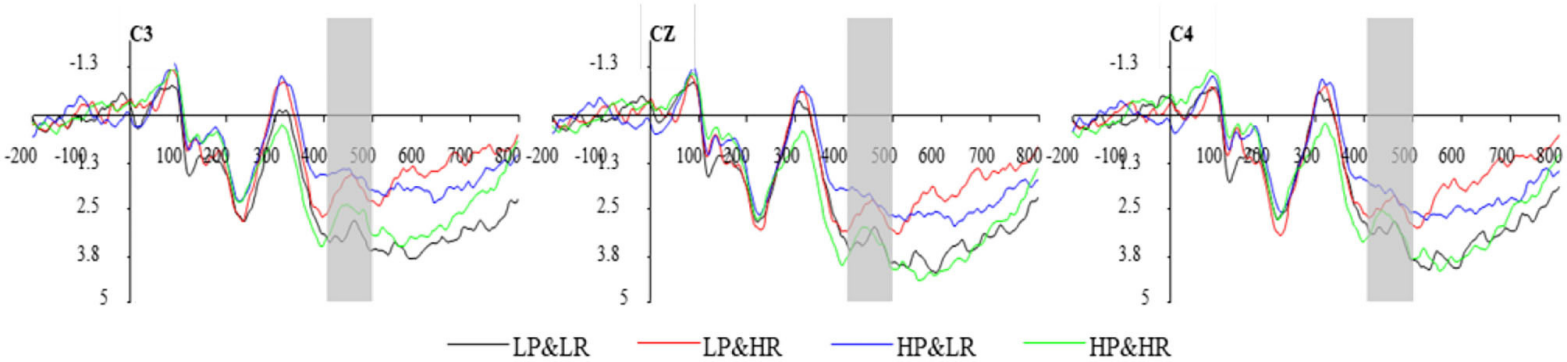
The average waveform of N400 component at C3, Cz, and C4.

As an important component of ERP, N400 locates mainly in the frontal and central areas of the brain. It is an ERP component that reflects information conflicts. A 2 (consistency: consistency and conflict) x 3 (central area electrodes: C3, Cz, C4) repeated measurements ANOVA was performed on the N400 component amplitudes in the 400–430 ms time window (Figure 7). When the review party's professionalism and popularity are in a consistent situation, the condition is defined as an agreement (HP&HR and LP&LR), otherwise conflict (HP&LR and LP&HR). The results show that the impact of consistency is not significant [*F*_(1,13)_ = 0.121, *p* = 0.7 33], the impact of conflict is not significant [*F*_(1,13)_ = 0.015, *p* = 0.664]. Therefore, in the decision conflict cognition stage of this study, conflicting cues did not affect the amplitude difference of N400. The result does not support the H2 hypothesis.

#### 3.2.3. LPP

**Figure 8:**
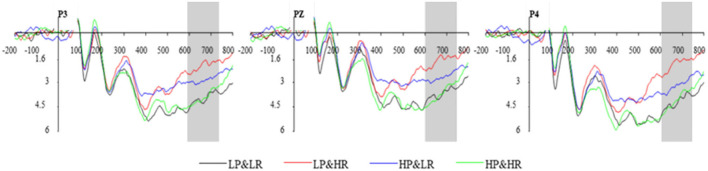
The average waveform of LPP component at P3, Pz, and P4.

**Figure 9:**
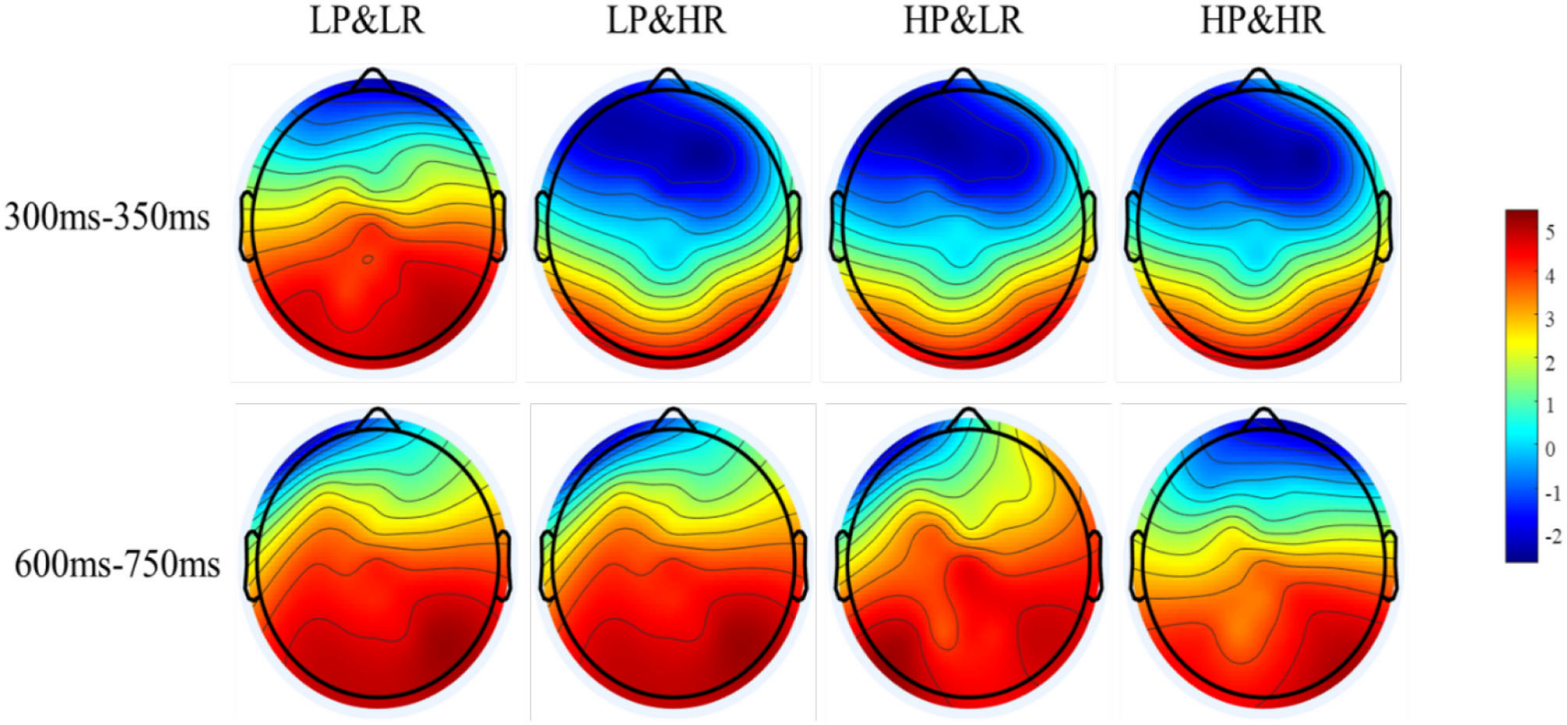
Brain voltage maps of N2 and LPP under different conditions.

In this experiment, the latency of LPP is approximately 600 to 750 ms. A 2 (HP vs. LP) x 2 (HR vs. LR) x 3 (frontal electrode sites: P3, Pz, P4) within-subjects repeated-measures ANOVA was performed on ERP amplitude between 550 and 700 ms (Figure 8). Results indicate a significant main effect of professionalism [*F*_(1,13)_ = 18.349, *p* < 0.001] and popularity [*F*_(1,13)_ = 6.214, *p* = 0.027]. The interaction effect between product rating and sales is significant [*F*_(1,13)_ = 12.191, *p* = 0.004]. Simple effect analysis shows that the LPP amplitude of low professionalism and low popularity (*M* = 2.287, *SD* = 0.632) is higher than the LPP amplitude of low professionalism and high popularity (*M* = 2.180, *SD* = 0.781). The LPP amplitude of high professionalism and high popularity (*M* = 4.505, *SD* = 0.983) is higher than the LPP amplitude of high professionalism and low popularity (*M* = 3.056, *SD* = 0.804). Therefore, in the final classification and evaluation stage, the difference in LPP amplitude under different conditions indicates that there is a process of overall evaluation and classification before the subjects decide to adopt. Thus, H3 is supported. The brain voltage map under different conditions is depicted in Figure 9.

## 4. Discussion

This study investigated the influence of two review parties' identity cues (professionalism and popularity) on consumers' review adoption intentions and the corresponding neural activity mechanisms using the ERPs technology. Subjects assessed their intentions to adopt reviews of products after presenting products and four different identity cue combinations.

### 4.1. Behavior

The behavioral results show that the professionalism and popularity of the review parties have a significant impact on the adoption intentions of the subjects, and the popularity has a significant effect on the response time. Compared with low-professionalism but high-popularity identity cues, high-professionalism but low-popularity identity cues incur higher adoption intentions. The cue diagnosis theory proposes that high-scope cues are more reliable and have a more significant impact on product quality evaluation, while low-scope cues are the opposite. Consumers perceive and diagnose review parties' identity cues with significant differences. High professionalism review parties' reviews receive higher degrees of recognition and willingness to adopt, while no significant recognition tendency is shown for popularity. It can be inferred that professionalism is a high-scope cue while popularity might be a low-scope cue. Compared with the 2-low-identity-cue situation (C1), the adoption rate is higher under the 2-high-identity-cue situation (C4). Meanwhile, consistent identity cues lead to faster decision-making, which is in line with the cue consistency theory (Xu et al., 2013). This is because consumers have different risk perceptions of review parties with different levels of professionalism and popularity. Review parties with high professionalism levels tend to be more authoritative, which can help consumers reduce perceived risk, increase trust, and raise the intention to adopt the reviews. In addition, consumers are vulnerable to review parties' popularities. Due to the influence of impulsivity and conformity, consumers tend not to pay more attention to the attributes of the product itself, nor to actively seek product information to help decision-making, but are highly dependent on high popularity opinion leaders. This is consistent with previous relevant research conclusions. Highly professional or highly popular leaders have a greater influence on consumers' decision-making intentions.

Previous studies have demonstrated that task completion time has a positive relationship with task difficulty and cognitive load. That is, the longer the response time, the higher the cognitive load and the greater the task difficulty (Ridderinkhof et al., 2004; Wang et al., 2016). Behavioral data show that the review party's popularity has a significant influence on response time. The average response times under the four conditions were significantly different. The average response time of C3 was the longest, and the average response time of C4 was the shortest, suggesting that C3 has the greatest decision difficulty while C4 has the lowest decision difficulty. The difference in response time indicates that subjects may have to make additional cognitive efforts when making decisions in C3. The adoption rate also supports this argument. The adoption rate under C3 is about 75% (0.755), indicating that subjects have a slight preference under C3 and are considering whether to adopt the corresponding reviews. In contrast, the adoption rate under C4 is close to 100% (0.955). Combined with response time data, this may indicate that subjects will consider adopting reviews under C4, so they spend the least cognitive effort under C4. The behavioral results suggest that subjects face higher cognition loads and greater task difficulties when making adoption decisions under low popularity conditions, which then indicates additional cognition efforts.

### 4.2. Event-Related Potential

In the early cognition stage of decision-making, N2 is closely related to risk perception. Research on risk and uncertainty decision-making has shown that the N2 component can be used as an ERP indicator for risky decision-making. Greater uncertainty and risky decision-making will result in an increase in the amplitude of N2 (Yang et al., 2007). Research by Wang et al. (2016) shows that low-rating products evoke greater N2 amplitudes. When consumers make purchase decisions, a low rating leads to greater perceived risk and decision-making conflicts and requires more cognitive effort. In this study, popularity has a significant effect on the amplitude difference of N2, and the amplitude of N2 under low popularity conditions (C1 and C3) is significantly higher than that of high popularity (C2 and C4). For consumers, low popularity means higher risks and uncertainties that may cause personal cognitive conflict and therefore requires more cognitive control that induces greater N2 amplitude. That is to say, compared with the professionalism of the review parties, the popularity is a higher risk indicator, which will be processed and perceived in the early stage by consumers, and the identity cues will be used as a diagnostic cue for the early perception of risk. Therefore, the results of this study can show that the popularity of the review party plays a very important role in the early decision-making stage.

In the later stage of decision-making, LPP is reported to be sensitive to evaluation and classification (Lin et al., 2018). When faced with positive (against negative) and high (against low) classification dimensions, it could elicit significant LPP amplitudes (Ito and Cacioppo, 2000). In this study, subjects were required to score the intentions to adopt reviews under different combinations of professionalism and popularity identity cues. The differences in ERP amplitudes are mainly determined by the valence of the stimuli. The analysis results show that both the professionalism and the popularity have a significant effect on the LPP amplitude differences. The average LPP amplitudes rank in the order of C4, C1, C3, and C2, which is also consistent with the adoption rate under the four experimental conditions. In the evaluation and classification stage, consumers have a tendency to use all cues to make evaluation judgments. When the classification of conditions is more in line with the standard category or closer to the expectations of the subjects, it elicits greater LPP amplitudes (Ito and Cacioppo, 2000). During the experiment, the subjects gradually build evaluation standards for review parties' identity cues. The standards are in line with self-expectations (Rotter, 1967; Nam et al., 2020). Subjects positively compare the review parties' identity cues against real-life scenarios, and the closer the identity cues are to the self-expectations, the stronger emotional arousal they could lead to Schupp et al. (2003). In this study, high-professionalism and high-popularity situations are more in line with the self-expectations of the subjects, while low-professionalism and low-popularity situations are less in line with the self-expectations of the subjects. Consistent identity cue conditions produce greater LPP amplitude. The behavioral results also support this argument. The subjects show a higher(C4)/lower(C1)adoption rate and faster response time when confronted with consistent identity cue conditions. In short, the difference in amplitude of LPP shows that there is a process of forming self-evaluation standards and evaluating classifications before finally adopting a decision.

## 5. Conclusion

This study exploits the ERPs technology to explore the influence of the differences in review parties' identity cues of professionalism and popularity on consumers' review adoption intentions from the perspective of the underlying neural mechanisms. Behavioral results revealed that consumers are more willing to adopt high-professionalism review parties' reviews. When facing low-professionalism review parties, consumers are affected by their popularities with relatively low adoption intentions. Consistent identity cues lead to shorter response times. The ERP results showed that in the early stage of risk perception, the popularity of the review party had a significant impact on the amplitude of N2, indicating that the popularity identity cue is a higher risk indicator. In the evaluation stage, the professionalism and popularity of the review parties jointly affect the adoption intentions. Consumers make comprehensive evaluations based on the consistency of identity clues and their self-evaluation standards (self-expectation), which significantly influence the amplitude of LPP. The findings of this study contribute to the marketing of product reviews and the review parties. It also enriches the theoretical framework and practical applications of neuromarketing.

### 5.1. Theoretical Contribution and Practical Significance

Previous studies on identity cues haven't investigated the joint influence of professionalism and popularity of review parties on consumers' review adoption intention. In this study, the two identity cues have been jointly examined with the ERPs technology. Unlike previous studies, we focus mainly on the adoption intention of reviews rather than purchase intention, which extends marketing research boundaries. Behavioral data shows that consumers show a higher degree of recognition and acceptance of highly professional reviewers' reviews, but they do not show a clear tendency to recognize their popularity. This study extends the cue diagnosis theory and cue consistency theory. Our findings indicate that professionalism belongs to high-scope cues and popularity belongs to low-scope cues. The use of ERPs technology reveals the underlying neural mechanisms of how the two identity cues of professionalism and popularity influence consumers' adoption intention of product reviews. It provides an understanding of the cognition processes of decision-making under a risky environment in an e-commerce scenario beyond traditional psychology that relies mainly on the recall of subject memories.

Our findings may be of practical guidance for e-businesses and review parties. E-businesses can benefit from publishing product reviews from high professionalism review parities that can increase consumers' review adoption intention and accelerate the purchase decision-making. E-businesses can also benefit from publishing reviews from review parties with significantly different popularities to help consumers with the product comparison process and make comprehensive decisions. For review parties, achieving high professionalism and high popularity should be set as their targets, especially for professional review agencies. The Matthew Effect of the Internet can help review parties reach business success. With high professionalism and high popularity identity cues, review parities can enjoy more opportunities to be published by e-businesses bringing them more fame among consumers.

### 5.2. Limitations and Future Directions

We acknowledge some limitations of this study that allows for opportunities for future studies.

- The ERPs technology relies on strict experiment environment and equipment settings to deliver accurate neural activity measurements. We removed lots of distracting environmental factors to limit the design of our experiment to a certain degree of abstraction of the real-life scenario. Future studies may benefit from adding environmental factors like the noisy surroundings and mobile devices to unveil the mechanism of the decision-making process in a distracting environment closer to real-life scenarios.
- The subjects recruited for our experiment were all students aged from 20 to 30. The selection ensures that the subjects have a good knowledge of e-commerce and e-marketing. However, it gives up the possibility of learning the decision-making patterns of subjects from a wider range of backgrounds. Future studies may benefit from recruiting a larger sample of subjects of a wider range of backgrounds.
- Our experiment uses electronic products (functional products) as the experiment materials. Future studies can benefit by extending the products to hedonic ones to examine whether product categories influence the review adoption intentions affected by review parties' professionalism and popularity.

## Data Availability Statement

The original contributions presented in the study are included in the article/supplementary material, further inquiries can be directed to the corresponding author.

## Ethics Statement

The studies involving human participants were reviewed and approved by Internal Review Board of the Academy of Modern Business Research Center of Zhejiang Gongshang University. The patients/participants provided their written informed consent to participate in this study.

## Author Contributions

LX and SW developed the study concept. FY contributed to the design. FW and YW collected the data and was supported by SW. SW and FW analyzed the data. LX provided the analysis tool and acquired the funding. FW wrote the first draft of the manuscript. All authors helped to edit and revise the manuscript and approved the final submitted version of the manuscript.

## Funding

This study received funds from the National Social Science Fund of China (No. 19BGL098), the Soft Science Research Program of Zhejiang Province (No. 2022C25042), and Key Research Institute (KRI) - Modern Business Research Center of Zhejiang Gongshang University (No. 2021SMYJ02LL).

## Conflict of Interest

The authors declare that the research was conducted in the absence of any commercial or financial relationships that could be construed as a potential conflict of interest.

## Publisher's Note

All claims expressed in this article are solely those of the authors and do not necessarily represent those of their affiliated organizations, or those of the publisher, the editors and the reviewers. Any product that may be evaluated in this article, or claim that may be made by its manufacturer, is not guaranteed or endorsed by the publisher.
